# F50. METACOGNITIVE REFLECTION AND INSIGHT THERAPY: A MULTICENTER RANDOMIZED CONTROLLED TRIAL

**DOI:** 10.1093/schbul/sby017.581

**Published:** 2018-04-01

**Authors:** Steven de Jong, Rozanne van Donkersgoed, Marieke Timmerman, Marije aan het Rot, Lex Wunderink, Johan Arends, Mark van der Gaag, Andre Aleman, Paul Lysaker, Marieke Pijnenborg

**Affiliations:** 1 University of Amsterdam; 2 Rijksuniversiteit Groningen; 3 GGZ Friesland; 4 GGZ Drenthe; 5 VU University; 6 Roudeboush VA Medical Center

## Abstract

**Background:**

Difficulties in metacognition, or the ability to think about thinking and feeling, form an impediment to daily life functioning for persons with a psychotic disorder. In the past years, our research team has undertaken a multicenter, randomized controlled trial to investigate the efficacy of a new intervention designed to assist persons with a psychotic disorder to improve their metacognitive functioning (Metacognitive Reflection and Insight Therapy; MERIT).

**Methods:**

After training thirteen therapists from seven mental healthcare institutes in the Netherlands, participants (n=70) with a DSM-IV-TR diagnosis of schizophrenia or schizoaffective disorder were included and randomized into either the MERIT condition or a treatment-as-usual condition. Persons randomized into the MERIT condition received 40 sessions of metacognitive psychotherapy, while persons in the control condition received care as usual. Measures of primary outcome (metacognition), secondary outcomes (empathy, depression, insight, stigma, social functioning, symptoms and quality of life) and control variables (neurocognition, premorbid IQ) were collected at baseline (pre), directly after therapy end (post) and at 6-month follow-up. After the follow-up measurement, research assistants were unblinded in order to conduct an interview with the participants regarding their experience of the therapy.

**Results:**

Multilevel intention-to-treat and sensitivity analyses demonstrated that in both groups metacognition had improved, with no significant differences between the groups (χ2 (1)=0.435, p=.51). At 6-month follow-up, however, participants in the MERIT condition demonstrated they had continued to improve on metacognition, while scores from the control condition dipped back down (χ2 (1)=3.763, p=.05). Gains mainly seemed to be on metacognitive Self-Reflectivity (χ2 (1)=10.295, p=.001). No effects were found on secondary measures in either condition.

**Discussion:**

During this presentation, we will discuss our findings and the therapy protocol, including a discussion of the clinical relevance of the current intervention, analysis of post-therapy interviews surrounding the participant’s experiences of the therapy, as well as practical limitations that were encountered during this five-year trial.

**Note:**

S. de Jong (speaker) and R.J.M van Donkersgoed are early career scientists, expected to defend their dissertations in 2018.

